# Can the South African Milestones for Reducing Exposure to Respirable Crystalline Silica and Silicosis be Achieved and Reliably Monitored?

**DOI:** 10.3389/fpubh.2020.00107

**Published:** 2020-04-07

**Authors:** Derk H. Brouwer, David Rees

**Affiliations:** ^1^Faculty of Health Sciences, School of Public Health, University of the Witwatersrand, Johannesburg, South Africa; ^2^National Institute for Occupation Health, National Health Laboratory Service, Johannesburg, South Africa

**Keywords:** exposure control, data analysis, under-reporting and diagnosis, grouping, bias

## Abstract

Silicosis and other respirable crystalline silica-associated diseases, most notably tuberculosis, have long been substantial causes of morbidity and mortality in South Africa. For the mining and non-mining industries, silicosis elimination programmes have been developed with milestones regarding reduction of levels of exposure to respirable crystalline silica (RCS) and targets regarding the date of eradication. The present paper explores the feasibility of achieving these targets by investigating the evidence that levels of exposure and silicosis incidence rates have declined by an appraisal of the methods for data collection and reporting. In the mining industry the silicosis elimination programme is supported by the development and advocacy of leading practices to reduce the exposure. RCS exposure data are routinely collected according to a Code of Practice (CoP) and the results are reported to the Mine Health and Safety Inspectorate. As the CoP and the actual workplace practices have been demonstrated to have some flaws, there is some concern about the accuracy of the actual exposure data and the data interpretation. The annually reported levels of exposure suggest a decline, however, the actual levels of RCS as well as the number of exposed workers, were not reported over the last few years. With regard to the silicosis incidence rates, a steady decline of new cases is reported. However, there is a risk of under-diagnosis and- reporting especially in former miners. In the non-mining industries, a systematic baseline of RCS exposure levels and silicosis incidence is lacking. The reporting by industries on assigning of the workforce to exposure categories seems to be fragmented and incomplete. Consequently, any evidence of progress toward achieving the silicosis elimination target cannot be documented. Both the silicosis elimination target and the exposure milestone are aspirational but are unlikely to be achieved. Nevertheless, the formal mining industry may get close. Exposure control interventions, especially in the non-mining industries, should be developed and implemented and pragmatic methods need to be put in place to identify sources of new silicosis cases for targeted intervention.

## Introduction

Silicosis and other respirable crystalline silica (RCS)-associated diseases, most notably tuberculosis, have long been substantial causes of morbidity and mortality in South Africa. Gold miners have borne the largest burden partly because of the high silica content of the gold bearing ores [silica content ranges from 9 to 39% and resulting silica percentages in airborne respirable dust vary from 5 to 57% (1)], and the social conditions associated with mining, such as migrancy and dormitory-style housing (2). In recent years, silicosis rates in currently employed gold miners have fallen (3) but, in the earlier part of this century, close to 20% of older in-service miners and about a quarter of former miners had silicosis (4). In the early 2000s, tuberculosis rates as high as 3,000 diagnosed TB cases per year for every 100,000 miners (3000/100,000/year) were recorded at gold mines (5). Even contemporary silicosis and active tuberculosis prevalence rates in gold miners coming to autopsy are concerningly high, i.e., 29.4, and 16.5%, respectively in 2017 (6). Autopsies have been dropped over the last decades from between 1,000 and 2,000 yearly over the period before 2010, up to about 500 in 2017 (6). In addition to the occurrence of disease, South African Gold mines and the workers in them have long been sources of data to define the relations between silica-rich dust and silicosis. A landmark early study used particle counts obtained by konimeters to show that there was a correlation between the amount of dust breathed and silicosis incidence (7). Hnizdo and Sluis-Cremer (8) plotted estimated cumulative RCS dust exposure (the product of intensity and duration of exposure in mg/m^3^ –years) against the risk of silicosis: silicosis risk started at about 3 mg/m^3^-years and rose rapidly after 7 mg/m^3^-years; 5% of miners developed silicosis at this dose. The standard modern measure of exposure—RCS (in the form of quartz)—was used by Churchyard et al. (4) to examine silicosis prevalence in gold miners exposed at a mean intensity of 0.053 mg/m^3^ (range 0–0.095) for a mean of 21.8 years. The authors stress that despite the RCS concentrations being < 0.1 mg/m^3^, ~19% of the miners contracted silicosis.

According to the Quarterly Workforce Survey in the third quarter of 2019, the formal mining industry employed 41,900 workers, whereas in the non-mining sectors were exposure to RCS is likely, i.e., construction, manufacturing, and agriculture, 3,979,000 workers are employed. The informal (non-agriculture) sector, defined as economic activity which takes place without a registered value added tax (VAT) number, is estimated to be 2,995,000 (9).

For historical reasons, the health and safety of workers in South Africa is governed by two occupational health and safety Acts, i.e., the Mine Health and Safety Act (no. 29 of 1996) (10) and the Occupational Health and Safety Act (no. 85 of 1993) (11). This means that, currently, two government departments (the Department of Mineral Resources and Energy, further referred to as DMRE, and the Department of Employment and Labor, further referred to as DEL, are responsible for implementing and enforcing the Acts. Consequently, both the mining- and the non-mining sectors have adopted different strategies to eliminate silicosis.

The Mine Health and Safety Council (MHSC) is a national public entity (Schedule 3A) established in terms of the Mine Health and Safety Act, No 29 of 1996, as amended^1^. The entity comprises a tripartite board represented by state, employer, and employee members under the chairmanship of the Chief Inspector of Mines. The MHSC has set milestones to eliminate silicosis (12): (a) by December 2024, 95% of all exposure measurement results will be below the milestone level for respirable crystalline silica of 0.05 mg/m^3^ (these results are individual readings and not average results); (b) using present diagnostic techniques, no new cases of silicosis will occur amongst previously unexposed individuals -; “previously unexposed individuals” are those unexposed to mining dust prior to December 2008, i.e., equivalent to a person who entered the industry in 2009.

For the non-mining industry, the DEL launched their National Programme for the Elimination of Silicosis (NPES) in 2004, setting the target for 2030, in line with the World Health Organization and International Labor Organization Global programmes (13). However, operationalization of the programme, for example, reporting interim exposure milestones, has not been reported. The current occupational exposure limit (OEL) for respirable crystalline silica (RCS) for both mining and non-mining industries is 0.1 mg/m^3^ TWA (8 h).

This paper explores the feasibility of achieving the milestones and silicosis targets set by the MHSC and DEL, respectively, by addressing issues related to: (i) the reliability of the collection, interpretation, and reporting of exposure data and the silicosis prevalence over the last decade, and (ii) evidence of associated decrease of reported exposure levels and silicosis cases.

## Materials and Methods

### Exposure Data

#### Data Collection and Reporting in South African Mining and Other Industries

The main objective of the measurements taken in South African mining and non-mining industries is compliance testing. Here, the selection of the workers to be sampled differs between the two sectors. The mining industry, governed by the MHSC Code of Practice, (14) uses the concept of homogeneous exposure groups (HEGs), based on activity areas, which have common intake air by ventilation. After defining the HEG, five or 5% of the workers in the HEG (whichever is larger) are randomly selected for sampling with regard to shift and occupation/job title. The frequency of sampling HEGs is determined by exposure categories (bands): A (exposures ≥ OEL), B (exposures ≥ 0.5 OEL and < OEL) and C (exposures ≥ 0.1 and < 0.5 OEL) bands. An exposure band is assigned to a HEG if the arithmetic mean (AM) and the 90th-percentile of the measurements for this HEG fall in the same exposure band. The sampling frequency or reassessment period is determined by the exposure category, and is quarterly, every six months and annually for the exposure categories A, B, and C, respectively.

In the non-mining industry, governed by the DEL, the Hazardous Chemical Substances Regulations (HCSR) (15) refers to the NIOSH—Occupational Exposure Sampling Strategy Manual (OESSM) approach (16) to include, in the sampled workers, those likely to have the highest exposure. The required level of confidence of selecting a worker or workers among the top 10 highest exposed workers depends on the type of OEL; this is 95% for crystalline silica which would require sampling all workers for groups with *n* ≤ 7 members. Measurements should be conducted every 12 months. Other recommendations by OESSM, e.g., the use of an action level which depends on the variance (e.g., GSD), is not followed since the measurements are not conducted by labor inspectors but by the Chief Inspector designated Approved Inspection Authorities (AIAs), entities that satisfy the DEL eligibility criteria to monitor regulatory compliance with workplace standards.

The measurement method is not mandated. However, the OEL (set by both the DMRE and the DEL) is for RCS dust, implying that the sampling would be according to consensus aspiration curves developed by International Standardization Bodies, e.g., ISO (17). In South Africa, the types of cyclones used are aligned with compliance to the respirable aspiration curves, in combination with the prescribed flow rates (18). Both X-Ray diffraction and Fourier-transform infrared spectroscopy (FTIR) are considered acceptable for the analysis of crystalline silica (19). Until 2018, the silica content in respirable dust in the mining industry was not based on individual personal samples, but on pooled filters (*n* = 5) of individual mixed cellulose (MCE) filters to make a potassium bromide (KBr) pellet for silica analysis using FTIR. The percentage silica of the pooled filters is then allocated to each filter. It was a means of increasing sensitivity of the analysis and reducing analytic costs. Nowadays most mines have switched over to using silver filters and XRD analysis of each individual filter.

In the mining sector, it is mandatory for the mines to report the personal exposure measurement data on a regular basis (depending on the exposure category of the HEG, but at least annually), according to a template. The report should contain information/data on the HEG, including occupations in the HEG and the number of workers per occupation; the pollutant code; the individual concentration values; the average of the HEG and the 90th-percentile; the percentage crystalline silica (based on pooled samples) and the calculated dose which is the average concentration of the HEG multiplied by the silica percentage; and the so-called pollutant index which is the ratio of the OEL to the “dose.” Finally, if more than one pollutant was measured, the Air Quality Index (AQI) of multiple pollutants should be calculated by dividing the dust concentration of each pollutant in the mixture by its OEL and adding the results.

For the non-mining sector, the DEL requires all industries handling, manufacturing and producing products that may cause exposure to silica dust to submit bi-annual reports which should include the following: number of samples taken and analyzed; maximum measured exposure level; and total number of employees exposed to silica dust and their allocation to the exposure categories or bands, similar to those used by the mining industry (20). For further analysis, we were able to retrieve in total 51 reports submitted to the Department over the period 2014–2017, covering four sectors.

#### Analysis of Exposure Data

Available data sets of personal (eight-hour) time weighted average [TWA (8 h)] concentrations of RCS dust from miners were analyzed using the Expostats tool^2^. Tool 1 was used for descriptive statistics and to determine the estimate of the 95th–percentile of the population of concentrations from which the samples were taken (21). Tool 3, enabling comparative analysis for two categories, was used to compare RCS-TWA (8 h) data of similar groups between years. RCS-TWA (8 h) concentrations, presented as arithmetic means (AM, μ) and standard deviations (SD, σ), were converted to geometric means (GM) and geometric standard deviations (GSD), according to the equations in Leidel et al.'s Occupational Exposure Sampling Strategy Manual provided (16).

(1) $$GM=\mu^{2}/\sqrt{\left(\mu^{2}+\sigma^{2}\right)}$$

(2) $$GSD=\exp\sqrt{\ln\left(1+{\frac{\sigma }{\mu^{2}}}^{2}\right)}$$

### Silicosis

#### Diagnosis

Typically, the diagnosis of silicosis is based on a history of sufficient exposure to RCS to cause the malady, imaging features (chest x-ray) consistent with the pneumoconiosis and clinical findings that do not suggest another disease. Sufficient exposure is difficult to define because very high exposure over a short period, even a few years, can be causative. (There is a debilitating and usually fatal form of silicosis termed acute silicoproteinosis which can occur following only months of exposure to extremely high concentrations of RCS, but this form is rare). For the common forms of silicosis, the dose of RCS dust inhaled over time is an important exposure variable to consider when assessing whether sufficient exposure has occurred (22). Dose here is the product of exposure intensity [usually an average RCS dust TWA (8 h) concentration] and duration of exposure in years. In workplaces with RCS at or below 0.1 mg/m^3^ silicosis usually arises after prolonged exposure, 15 to 20 or more years being the norm. The imaging features are easily recognizable to an experienced X-ray reader, provided they are not rendered atypical by associated tuberculosis, very high exposures, or other incidental or complicating conditions. RCS causes an increased risk of tuberculosis, so clinical features of this condition should be sought when evaluating individuals exposed to the dust.

#### Reporting

South Africa has several statutory provisions for the reporting of silicosis to the DMRE and DEL enforcement agencies. The MHSA compels the mine employer to ensure that an employee is under medical surveillance if the employee might be exposed to a hazardous substance, if there is reasonable evidence that such exposure may cause a disease or the occupational health practitioner recommends medical examination. The mine employer must report occupational diseases, including silicosis, to the Principal Inspector, within 30 days of the diagnosis. A template for reporting (the Health Incident Report) includes the individual miner's exposure history. Additionally, every occupational medical practitioner at a mine should compile an annual report that covers employees at that mine and analyses the employees' health status based on the medical surveillance records, without disclosing the names of the employees (Section 16, MHSA). The annual medical report (AMR) must be given to the employer, who must, in turn, send the report to the medical inspector. The DMRE's Mine Health and Safety Inspectorate reports on the submitted AMRs, annually.

For the non-mining industries, the Hazardous Chemical Substances Regulations are the main statutory vehicle for medical surveillance. The Regulations obligates employers to ensure medical surveillance in similar clauses as the MHSA. In addition to workplace medical surveillance, the medical practitioners are obligated to report work-related diseases to the Chief Inspector in terms of Section 25 of the Occupational Health and Safety Act (no. 85 of 1993). Details of the employer and occupational history are required. Submission of claim for a worker's compensation for an occupational disease, e.g., silicosis, is the joint responsibility of employees and employers according to the Compensation for Occupational Injuries and Diseases Act (COIDA) (Section 65). Cases that are eligible for compensation are registered at the Compensation Fund, which is established by COIDA (No. 130 of 1993) and its amendments.

## Results

### Exposure Data

#### Reliability of Collected Data

A number of issues are associated with the reliability of exposure data. First, the effectiveness of the surveillance or oversight of the sampling can be questioned, especially in underground mines. Common practice is that the pump and sampler are attached to the worker in the lamp room and, at the end of the shift again collected. During the shift, the oversight is transferred to shift bosses/foremen who are not in proximity to all the workers wearing. It is thus unclear if and when the sampler is actually worn. Smart pumps with accelerometers (23) can provide additional information to evaluate whether the pump is worn, but these are not currently in use. Second, most of the samplers used are manufactured in South Africa and their design is based on the original Higgins-Dewell (HD) type of cyclone. Recently reported results of an evaluation study of currently used South African gravimetric samplers, compared against the original UK SIMPEDS, showed that the South African samplers have a systematic 50% cutoff size (D_50_) sampling bias as high as 59% against the size-selective curve (18). Similarly, under controlled laboratory coal dust test conditions, measuring the same coal mine dust level in a chamber, the South African and UK standard SIMPEDS samplers recorded concentrations of 7.87 and 6.71 mg/m^3^, respectively, which aligned with the sampling bias. The author stated that the differences can be attributed, in part, to the “un-auditable” inherent design and manufacturing quality, or unverifiable data on the size-selective sampling curve. Moreover, humidity (24) and air density (25) in the deep mine conditions, and thus the Reynolds number, deviate from surface conditions. In combination with the practice of on-surface calibration, this provides theoretical reasons for doubting compliance of the sampling with the respirable convention (26, 27). Third^*^, the practice to pool filters for RCS analysis to reduce the number of non-detects, as well as to reduce costs, may have masked the true variation and associated misclassification of individual exposure concentration results.

#### Data Interpretation

With regard to the data interpretation and statistical analysis, the current practice in the South African Mining Industry (SAMI) has potential for interpretation flaws. First, the total sampling time is used to average the collected RCS mass on the filter as a TWA (8 h) concentration. Quite often, the sampling time exceeds eight hours, however, as this period includes the time to travel to and from the actual work location to the lamp room, during which RCS concentration may be low. The whole-shift sampling strategy masks the contributions of certain work-activities to the overall TWA (8 h) concentration, which would be relevant for controlling exposures. Second, (underground) miners have a relatively high level of exertion (28) which affects the deposition efficacy and location of the inhaled particles in the respiratory tract. Thus, their TWA (8 h) personal breathing zone concentration may not be directly comparable to the OEL, since the conditions deviate from the standard assumptions taken into account for setting an OEL, namely, that of moderate physical demands (29). Third, the statistical analysis of the collected data described in MHSC CoP is limited; It describes only the determination of whether the mean and empirical 90^th^ percentile (of observed data points) of the HEG fall in the same classification band, i.e., A (exposures ≥ OEL), B (exposures ≥ 0.5 OEL and < OEL) and C (exposures ≥ 0.1 and < 0.5 OEL) (14).

This approach can be criticized since it implicitly assumes a normal distribution of the RCS dust concentrations and ignores the fact that the data represent only a sample of the true distribution of the population of concentrations. In addition, the acceptance of the 90th percentile as the upper limit of the classification band implicitly means that, for classification band B 10%, exceedance (of actual observations) above the OEL is accepted. Ongoing research demonstrates that this approach substantially underestimates the potential of exceedance of the OEL compared to internationally accepted strategies, e.g., CEN (30), and professional bodies like BOHS/NVvA (31), where the point of departure is that the sample is taken from a lognormal distribution and 5% exceedance is acceptable. Moreover, in these approaches the uncertainty is explicitly addressed by using the 70% upper confidence limit on the exceedance fraction to 5%, which is equivalent to the criterion that the 70% upper confidence limit of the 95th-percentile should be below OEL (32).

**Figure 1:**
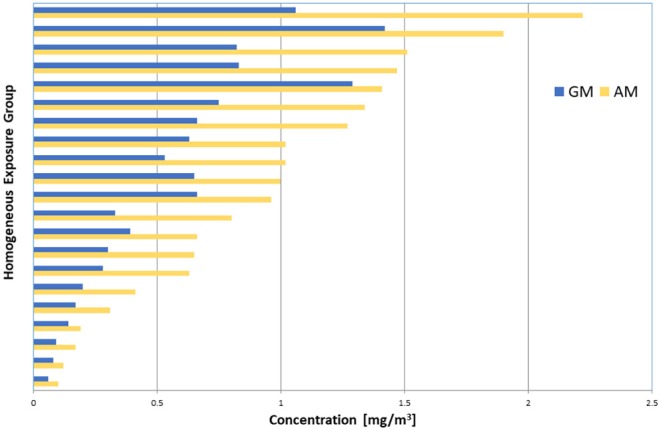
Plot of the AM (yellow bars) and GM (blue bars) of personal coal dust concentrations of 21 HEGs with > 15 data points. Data (*N* = 684) were collected from 6 coal mines in 2015.

**Figure 2:**
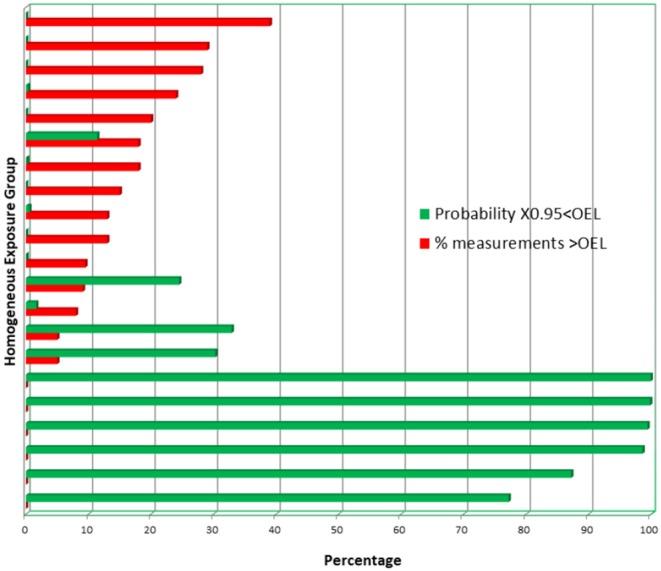
The percentages of individual TWA (8 h) coal dust concentrations that exceed the OEL (red bars). The green bars reflect the probability that the 95th percentile of the estimated distribution of the population from which the samples have been taken is below the OEL.

Potential misclassification of HEGs to exposure bands, especially an incorrect allocation to exposure classification band B, can be illustrated by an example of 21 HEGs. Each of the HEGs has more than 15 gravimetrically-determined TWA (8 h) coal dust concentrations, collected from six South African coal mines in 2015. The total number of TWA (8 h) personal dust concentrations was 684, and the 2015 OEL for coal mine dust (silica content <5%) was 2 mg/m^3^. The AMs and GMs of the HEGs are presented in Figure 1. In Figure 2, for each HEG, the percentages of individual TWA (8 h) concentrations exceeding the OEL are plotted, as well as the probability that the 95th percentile of the estimated population distribution will be below the OEL. According to the MHSC approach, 10 HEGs demonstrate “overexposure” (exposure band A) since ≥ 10% of the observations exceed the OEL. According to the BOHS/NVvA approach, six HEGs demonstrate “compliance” since the probability that the 95th percentile is below the OEL is higher than 70%. Hence, there is agreement between the two approaches for the 16 HEGs. For the remaining five HEGs, the two approaches show disagreement; According to the MHSC approach the exceedance of the OEL by individual TWA (8 h) concentrations was below 10%, hence, these HEGs would be classified as band B. However, according to the BOHS/NVvA approach that the compliance criterion is met for these HEGs has only a probability of about 30% and lower, which indicates a risk of overexposure, hence a classification as band A. In practice, it means that large groups of workers may not be identified as potentially being at risk.

It is unclear whether the non-mining industry adheres to the Hazardous Chemical Substances Regulations and the associated OESSM approach regarding the required frequency of measurements (yearly). The total number of reports submitted to DEL suggests, however, that this is not strictly followed. There is no evidence that any statistical analysis of the RCS-TWA (8 h) concentrations are deployed.

#### Evidence of Exposure Reduction

To address health and safety concerns and accelerate the progress toward achieving zero harm and finding sustainable solutions for the attainment of the occupational health and safety milestones, the then Chamber of Mines (now the Minerals Council) and its social partners, Government and labor established the Mining Industry Occupational Safety and Health (MOSH) in 2003 to focus on the adoption of leading practices identified in so-called source mines for hazard reduction with emphasis on the implementation of dust controls^3^. The claimed effectiveness of reducing RCS concentrations by engineering controls were, on average, ± 90% for fogger dust suppression systems (1), ± 65 % for footwall and side wall wetting (33), ± 70% for multi-stage filtration systems (34), and ± 50% for winch covers (35). More recently, the continuous personal dust monitor (CPDM) was introduced as leading practice in MOSH, reflecting international developments (36), enabling miners to immediately take actions to reduce their exposure, for example by altering their location. The extent of the roll-out of the leading practices over the mining sector in South Africa is not clear; however, Minerals Council members volunteered to provide information on their performance with regard to the RCS milestone. Similar initiatives in the non-mining sector do not exist.

With regard to the reduction of workers' exposure to RCS, the reporting by the governing departments should be the first source of information from which to draw conclusions. Until 2014 the DMRE's Mine Health and Safety Inspectorate (MHSI) reported the results of the individual measurements to monitor the progress meeting the former milestone, i.e., by December 2008, 95% of all exposure measurement results had to be below the OEL for RCS of 0.1 mg/m^3^. In 2014 there were 7,400 samples below the 0.1 mg/m^3^ out of a total of 7,842 samples taken (37). This resulted in a 94.6% compliance, which equates to an improvement of 2.6%, when compared to 2012 at 92%. Unfortunately, the more recent annual reports are not unambiguous. First, the personal RCS dust exposures are not directly reported, but, are “hidden” in the AQI, which is presented as A, B or C exposure classification. Second, the data are presented as the percentage of the total number of HEGs from which samples were collected. Consequently, they do not reflect the total percentage of exposed employees in the mining industry, since neither the number of HEGs nor the number of workers designated to the HEGs are provided. Over the years, there has been a steady decline of production and employment in gold mining; in 2013 the gold mines employed 131,738 direct employees but this had decreased to 101,085 in 2018. These figures represent 25.7 and 22.3%, respectively, of the total workers employed in the SAMI (38, 39).

Nonetheless, if we consider the AQI as a proxy for the RCS exposures, an overall decrease can be observed in the percentage of HEGs classified as category A (≥ OEL) from 6.7% in 2012 to 3.52% in 2017 (37, 40), for the SAMI (all commodities). For gold mines, this percentage dropped from about 8% in 2013 to 3.84% in 2017. More interesting from the perspective of the current milestone value, are the figures of the HEGs classified as C (< 0.5 OEL), which can be a proxy of 0.05 mg/m^3^ RCS. For all commodities, the percentage of HEGs falling in the exposure band C show a sharp increase from 65.7% in 2012 to 79.07% in 2017. For the gold mines, however, the increment was less substantial, i.e., from about 62% in 2013 to 66% in 2017.

Ancillary information can be retrieved from data available in the public and private domains. With regard to RCS exposure of gold mine employees in South Africa, both Churchyard et al. (4) and teWaterNaude et al. (41) reported exposure data of the same group of workers recruited in a single gold mining company in the North West province of South Africa, for epidemiological studies. The RCS-TWA (8 h) personal dust concentrations of the study participants (*n* = 497), collected in 2000, were similar to those of a subset (*n* = 715) selected from a dataset of routinely collected samples of some 7,700 records, i.e., AM = 0.048 mg/m^3^ and SD = 0.072 mg/m^3^, recalculated to GM = 0.026 mg/m^3^ and GSD = 2.72, respectively. teWaterNaude et al. (41) also reported some data per occupational group.

Exposure data of similar occupational groups were, more recently, routinely collected from three gold mines in North West province (42) and two gold mines in the Free State province (43) (Table 1). A direct comparison across time is not feasible, since between mine differences of the RCS concentrations are substantial; this is assumed to be associated with the silica content of the ore body (31), which can range from 9 to 3 9% (1, 44). The silica content in the two Free State mines (B and C) was particularly high, at 38% and 25% (43).

**Table 1:** Overview of personal RCS-TWA (8 h) concentrations for two occupations in South African gold mines.

**Job title**	**Rock Drill Operator**						**Scraper Winch Operator**			
**Mine**	**A**	**B**	**C**	**D**	**E**	**F**	**A**	**D**	**E**	**F**
Year	2000^1^	2008^2^	2008	2010^3^	2010	2010	2000	2010	2010	2010
N	94	31	28	13	18	29	75	20	20	18
Median		0.094	0.04	0.014	0.048	0.024		0.013	0.045	0.03
AM	0.075	0.142	0.05	0.03	0.053	0.055	0.065	0.024	0.127	0.031
GM		0.101	0.038	0.016	0.051	0.027		0.015	0.055	0.021
GSD		2.42	2.22	3.59	1.8	3.5		2.85	3.69	2.56
P95		0.441	0.148	0.135	0.117	0.208		0.091	0.453	0.096

1 *Data reported by teWaterNaude et al. (41)*.

2 *Data reported by Kemsley (43)*.

3 *Data reported by Kesilwe (42)*.

Table 2 shows an overview of RCS-TWA (8 h) personal dust concentrations collected in two gold mines located in the North West province. For three different occupations (job titles) the descriptive statistics are presented for two different years, which enables a comparison. For both the rock drill operators and the loco operators, a slight, but not statistically significant, decrease in the dust concentrations across time is observed. For the scraper winch operators, the RCS-TWA (8) concentration seems to be significantly decreased in 2015 for mine II but, in contrast, seems to be slightly increased in mine I. However, the relatively low numbers for mine II and the high GSD (3.15) in 2015 contributes to this finding. In addition, a decrease in exposure levels of loco operators in mine I is not evident; however, the number of observations in 2015 is only 30% of the number in 2010. Overall, the proportions of observations exceeding the proposed OEL milestone of 0.05 mg/m^3^ is in mine II across the three job titles were below 5% in 2015. In contrast, the corresponding figures for mine I show proportions ranging from 17 to 40%.

**Table 2:** Summary of RCS-TWA (8h) personal dust concentrations (mg/m^3^) for three occupations in gold mining.

**Job title**	**Rock drill operator**				**Scraper winch operator**				**Loco operator**			
**Mine**	**I**		**II**		**I**		**II**		**I**		**II**	
Year	2010	2015	2010	2015	2010	2015	2010^*^	2015^*^	2010	2015	2010	2015
N	79	59	40	47	17	34	128	230	300	110	153	149
Median	0.04	0.033	0.018	0.02	0.032	0.038	0.013	0.015	0.033	0.046	0.02	0.016
AM	0.045	0.035	0.025	0.022	0.033	0.039	0.023	0.018	0.049	0.045	0.024	0.019
GM	0.033	0.027	0.017	0.018	0.027	0029	0.019	0.014	0.028	0.034	0.018	0.016
GSD	2.27	2.45	2.62	1.95	2.07	3.15	1.94	2.02	2.78	2.62	2.17	1.93
%> 0.05 mg/m^3^	37	17	12	2.1	18	26	4.7	3	33	40	4.6	3.4

* *Significant difference*.

**Figure 3:**
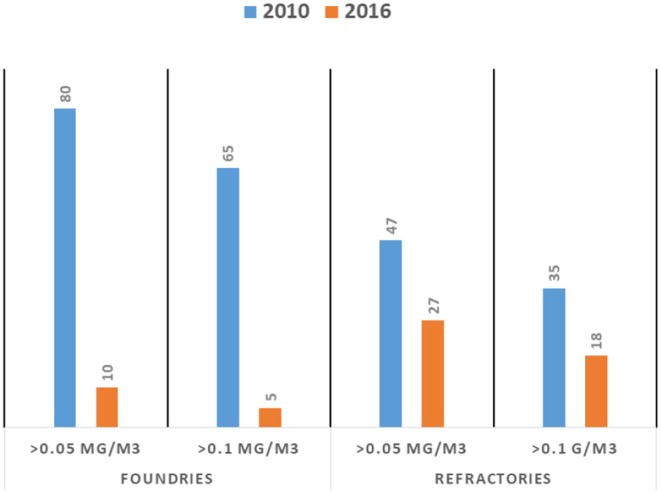
Comparison of percentage of personal respirable crystalline silica TWA (8 h) concentration measurements exceeding 0.05 and 0.1 mg/m^3^ for foundries (left panel) and refractories (right panel). The 2010 data are reported by Khoza et al. (45) and the 2016 data are retrieved from AIA reports (*N* = 20). The number of data points for the foundries and refractories are 54 and 57, and 41 and 44, respectively.

For the non-mining sector, the DEL does not publish exposure data in their annual reports; however, some data can be retrieved from the reports submitted by industries to the DEL (Table 3). In the period 2014 to 2017, of the 51 report retrieved, the percentage of reports with missing data and/or inconsistent information/data is relatively high (35%). The data suggest that overall about 25% (529) of the workers exposed to RCS dust (2073) would have been measured, however, it is not clear how they have been selected. The ranking of the sectors according to the proportion of workers with RCS-TWA(8h) concentrations > 0.1 mg/m^3^, i.e., ceramics, foundries, refractories, and construction, is similar to the ranking based on actual TWA (8 h) concentration measurements collected in 2010 for a research project (45). If the TWA (8 h) levels from that study are compared to the actual measurement data for foundries and refractories, as reported by AIAs in 2016, a substantial decrease in the proportion of observations exceeding 0.1 mg/m^3^ (the current OEL) and 0.05 mg/m^3^ can be observed of (Figure 3). However, in 2016, 10% (foundries) and 27% (refractories) still had measurements that exceeded 0.05 mg/m^3^.

**Table 3:** Summary of reports (2014–2015 *n* = 4) and (2016–2017 *n* = 47) submitted to DEL according to the reporting sheet template.

**Sector**	***N* reports^a^ (%)^b^**	***N* measurements**	**Representing *N* workers**	***N* workers ≥ 0.1 mg/m^**3**^ (%)**	***N* workers ≥ 0.05 mg/m^**3**^ (%)**
Foundries	12 (80%)	187	824	113 (14%)	222 (27%)
Refractory	16 (53%)	206	635	110 (17%)	420 (66%)
Construction	17 (68%)	57	93	0 (0%)	0 (0 %)
Ceramics	6 (75%)	79	521	93 (18%)	152 (29%)
Total	51 (65%)	529	2073	316 (15%)	767 (40%)

a *Reports that were considered reliable, e.g., no inconsistencies, no missing data etc*.

b *Percentage of total reports submitted to DEL*.

### Silicosis

#### Diagnostics and Monitoring

Essentially, the diagnosis of silicosis requires an experienced doctor to recognize the imaging features, confirm that enough exposure has occurred to RCS to cause silicosis—this requires knowledge about high risk jobs—and a clinical evaluation to exclude other conditions; tuberculosis and sarcoidosis are especially important in South Africa. Importantly, exclusive reliance on the chest x-ray for the diagnosis is to be discouraged. The above mentioned parameters indicate a high risk of under-diagnosis by non-experienced, non-occupational physicians.

Monitoring the progress toward achieving the silicosis milestone and target presents several difficulties. Silicosis can manifest radiologically long after the last exposure has occurred. A cohort study of South African gold miners showed that 57% developed radiological signs of silicosis after mining exposure had ceased; on average, 7.4 years after leaving mining (8). Cross-sectional surveys of currently employed workers cannot verify the absence of silicosis occurring in an industry; follow-up of former workers is required – an onerous task in an under-resourced setting in which migrant workers may disperse to rural labor-sending areas. A national register of incident silicosis cases would be an attractive solution, sustainable reporting might be an issue.

For example, South African health workers have been shown to poorly sustain reporting of cases: the SORDSA national registry of work-related respiratory diseases had 393 contributors in 1996 but only 27 in 2002, despite active encouragement to report diseases (46). An alternative to relying on doctors to report cases is to systematically scrutinize hospital discharge records, a method shown to be cost-effective in areas of the USA (47). However, in South Africa, patients with uncomplicated silicosis are rarely admitted to hospital, so very few cases would be identified in this way. Workers' compensation claim data could be a valuable source for monitoring occupational disease occurrence, but under diagnoses and underreporting of occupational diseases means that the absence of silicosis claims do not imply that the disease has been eliminated.

#### Evidence of Reduction

The DMRE's Mine Health and Safety Inspectorate (MHSI) reported a steady decline in new silicosis cases over a five years period (2012–2017). Based on the submitted AMRs, the absolute number dropped from 1 420 in 2012 to 652 in 2017, with associated incidence rates of 2.41 and 1.25 cases per 1,000 employees, respectively (Table 4). The contribution of the gold mines to the number of reported cases was more or less stable over this period, i.e., about 80%. The completeness of these figures can be questioned, however, if compared to figures released by the Occupational Lung Diseases Working Group, a collaborative initiative of the six largest mining groups in SA (https://www.oldcollab.co.za/resources/in-the-news/2018). The group reported that silicosis diagnoses had dropped by 24% from 853 cases in 2015 to 635 cases in 2016 on the four gold mines in the group^4^. The MHSI, however, reported 543 cases in 2016 for all gold mines.

**Table 4:** Reported cases of silicosis as reported by DMRE/ MHSI over period 2012–2017.

**Year**	**2012**	**2013**	**2014**	**2016**	**2017**
Annual medical reports (N)	708	761	836	902	975
Employees covered by AMRs (N)	590352	576716	Not reported	541519	521358
Silicosis cases (N)	1420	1430	1063	635	652
Proportion gold mines (%)	78.5	81.4	78.7	85.5	77.6
Cases per 1000 employees (N)	2.405	2.479	-	1.173	1.25

To our knowledge, no formal evaluation of the completeness of occupational disease reporting has been performed for the non-mining industries. For example, there are no published annual reports from the inspectorates, regarding the number of diseases reported, the causative workplaces, or remedial actions taken to reduce exposures and prevent future cases. Unfortunately, the annual reports of the Compensation Fund do not provide any information on the number and type of occupational disease cases submitted or compensated (48). Therefore, no evidence of reduction of silicosis can be retrieved from these sources.

## Discussion

### Exposure

For both the mining and the non-mining industries in South Africa, the relevant governing departments have developed a programme to eliminate silicosis in the near future. For the mining industry, this has been operationalized by the introduction of a milestone regarding the levels of RCS-TWA (8 h) concentrations and another to ensure that there are no new cases of silicosis in those first exposed after 2008. For the non-mining industry, such concrete performance indicators are lacking, other than that no new cases of silicosis should arise after 2030. This paper explores the feasibility of programmes with regard to the decrease in the levels of RCS-TWA (8 h) and silicosis, and the monitoring thereof.

For the formal mining industry, there is evidence that the levels of RCS-TWA (8 h) have decreased over the years; however, the present DMRE-MHSI reporting is too veiled to draw firm conclusions with regard to achieving the exposure milestone. An in-depth analysis of the “raw RCS exposure data” reported to the DMRE over the last five years and captured in their electronic information system is needed to allow critical scrutiny of the progress toward achieving the milestone. The scattered information collected in the present paper only provides an impression of descriptive statistics of RCS-TWA (8 h) concentrations in different years. The datasets were generated for specific study objectives and do not provide sufficient information for a time trend analysis, as the variation of RCS-TWA (8 h) concentrations is high, and within-mine data sets across time are difficult to access.

No exposure milestone has been set for the non-mining industry. In the present paper, we assumed that such an exposure milestone could be similar to that for the mining industry, i.e., 0.05 mg/m^3^. It is acknowledged, however, that similar to the setting of OELs, an exposure milestone would be a consensus value where technical and economic feasibility would play an important role in moderating a health-based limit value to prevent silicosis (49).

Only fragments of information could be retrieved with regard to past and present exposure to RCS dust for some sectors in the non-mining industry. Moreover, the representativeness of the relatively few measurements for an entire sector can be questioned. For example, in a study by Khoza et al. (45), specific activities and or occupations in the construction sector were not included, and these have been reported in the literature as having high exposures (50, 51). Hence, for the non-mining industries, it is even harder than for the mining-industry to draw conclusions regarding a decrease of RCS-TWA (8 h) concentrations across time, if any. The few data that are available (Table 3 and Figure 3) indicate, however, that an impressive percentage of high risk workers (on average, 40%) are currently exposed to RCS-TWA (8 h) concentrations above 0.05 mg/m^3^.

Clearly, the monitoring of both the exposure levels and the achievements to reduce occupational exposures to RCS dust, and the reporting, have room for improvement. Since the OEL and the current exposure milestone are based on personal RCS dust mass concentrations, active sampling using pumps and cyclones and off-line analysis will still remain the standard. Recently, methods have been piloted that enable direct-on-filter detection of quartz, including portable FTIR and consequently allow immediate response by the worker or the management in case of high exposures (52–54). Other metrics, such as number concentration and surface area concentration, might be more appropriate for health-relevant exposure assessment (55). The health-relevance of exposure to (ultra) fine crystalline silica particles was recently reported by Ophir et al. (56) in a study of exposure to artificial stone dust, containing a high percentage of free crystalline silica (51). The authors found a strong association between the presence of ultrafine particles in induced sputum and the level of inflammatory cytokines (57). Chubb and Cauda (58) investigated the particle size distribution of crystalline silica in gold mine dust and reported that the composition of (experimentally resuspended) South African test gold mine dust was dominated by submicron particles, i.e., count median diameter of 0.59 μm and mass mean diameter of 3.92 μm. Characterization of the particle size distribution, either as aerosols or as particles deposited on collection filters by laser light scattering, has been introduced in South Africa only recently. Direct measurement of the lung deposited surface area (LDSA) dose is currently not performed; the appropriate devices were introduced a few years ago and only recently deployed in the USA to assess exposure in mining operations (58). Since the contribution of submicron particles to the respirable mass concentration is relatively small (59), the relevance of the smaller silica particles may be underestimated.

From a statistical point of view, the interpretation of the measurement data needs improvement. Despite the development of new generation (relatively) cheap real-time monitors and sensors (60–62), compliance testing of the entire workforce or group in a specific exposure scenario is based on a relatively small number of samples. To address temporal and spatial variability and uncertainties, a probabilistic (Bayesian) exposure assessment provides a more realistic view of the population's exposure (21). It may imply that, in cases with <5% of the individual RCS-TWA (8 h) measurements below 0.05 mg/m^3^ (the mining industry's milestone), there might still a probability that the 95th percentile of the population distribution will exceed that concentration. Hence, some individual workers may be at higher risk compared to what the group average exposure level would indicate.

The current reporting of respirable crystalline exposure levels by the responsible departments is insufficient to monitor performance with regard to achieving the milestones and the objective of the silicosis elimination programme. The mining sector, in particular, has abundant data which would allow for appropriate monitoring and reporting progress to achieving of milestones. For the non-mining sector, the lack of data is the biggest stumbling block to providing an overview of RCS dust exposures, especially since it includes numerous small and micro-sized enterprises. The DEL is planning to employ many hundreds of new inspectors which could enhance the capacity to conduct measurements (Personal communication, Chief Inspector, Department of Employment and Labor, 2019). Clearly, the presently deployed reporting sheet that requires to report only the highest measured RSC-TWA (8h) needs to be revised, so the Department should be able to capture all measurement data.

Supportive actions to reduce exposure, such as those initiated in the mining industry through the MOSH learning practice, will be pivotal for the non-mining industries. The traditional approach with regard to exposure control, i.e., substitution, emission control, e.g., by wetting, ventilation and worker training, and personal protection, can be used to develop cost-effective interventions (63). The diversity of the industries and associated exposure scenarios will, however, require tailor-made exposure control solutions.

### Silicosis

Both the DEL's target of silicosis elimination by 2030 and the MHSC's milestone of no new cases in those first exposed after 2008 are laudable, but almost certainly unachievable. Additionally, ascertaining whether they have been attained will be very difficult.

A cardinal reason that South Africa will fall short of its silicosis elimination aspirations is that the current RCS OEL of 0.1 mg/m^3^ is not protective against silicosis; nor is the lower halved target concentration of 0.05 mg/m^3^, which is the MHSC milestone. The USA's Occupational Safety and Health Administration (OHSA) has estimated the risk of silicosis after 45 years of exposure at 0.1 mg/m^3^ to range from 60 to 773/1000 workers, depending on the nature of the industry and other moderating factors; at 0.05 mg/m^3^ the risk is estimated to be 20 to 170/1000 workers (64).

The lack of full protection afforded by these standards would be immaterial if South African workplace RCS concentrations were consistently below 0.05 mg/m^3^, but they are not, as shown by the data presented in the figures and tables in this paper. The risk of chronic silicosis—the most common form of the pneumoconiosis—is largely determined by cumulative exposure, i.e., a function of concentration and duration of exposure (65). This makes realizing the goal of no new silicosis cases in mining in those first exposed after 2008 problematic. When the milestone was set, it was well-known that RCS exposures in many settings exceeded 0.05 mg/m^3^, hence the exposure milestone of “By December 2024, 95% of all exposure measurement results will be below the milestone level for RCS of 0.05 mg/m^3^.” So, even if this ambitious exposure milestone is realized by 2024, some mine workers will have accumulated 16 or more years of RCS exposure; For example, at 0.075 mg/m^3^, the cumulative exposure will be 1.2 mg/m^3^ -years, a figure shown to be associated with silicosis risk in miners (22).

Perhaps the country should be pragmatic about monitoring the attainment of silicosis elimination: being able to confidently assert that no cases are occurring is unrealistic. First, the current silicosis class action case against the gold mines has shown that when workers leave employment at a mine and return to their rural homes, the onset of silicosis may not be detected by under-resourced local health services^5^. Second, there might be an underreporting, as demonstrated by the discrepancy between figures issued by the MHSI and the Occupational Lung Disease Working Group. This might be partly due to the large number of workers being employed by labor brokers or contractors. Various estimates suggest that up to one-third of an operating mine's total labor force may be employed in this manner. Disease in these workers is unlikely to be picked up by the mine health surveillance system and, consequently, will be off the radar when it comes to evaluating progress on the milestone targets^6^.

It must be realized that everything mentioned above is valid for the formal mining sector. When the large-scale gold mines in the Witwatersrand basin were dismantled in the late 1990s, many redundant workers turned to illegal mining in the region to create a living (66). It is clear that, like other workers in the informal sector, the growing number of “zama-zama” miners is not caught by a health surveillance or compensation system, so new cases of silicosis—even if diagnosed—will not be reported.

Nonetheless, using targeted systems to determine whether new cases are occurring should be feasible. The autopsy findings of deceased miners provide an example. In South Africa, all deceased miners who have done risky work (dusty work, in effect), even for a short period, are entitled to an autopsy to identify compensable diseases, including silicosis. Use of the service is far from complete (67)—only 801 autopsies were performed in 2017—but the findings are of considerable value; 150 cases of silicosis were found in the 801 cases examined in 2017 (6). Monitoring the occurrence of the pneumoconiosis in those first exposed after 2008, although incomplete, would provide insight into progress of achieving the mining milestone. Reporting by a small number of interested doctors in selected settings, for example, occupational medicine clinics and pulmonology units, may provide the same insights as the State autopsy findings for the DEL: are some cases still occurring, and if so, in which workplaces and industries? Compensation data, although incomplete, will still provide evidence that silicosis is occurring, even if the absence of claims cannot be used to infer that it is not. Targeted periodic surveys of long-service workers in particularly high risk jobs and workplaces, such as silica quarries, high dust-generating construction tasks, and abrasive blasting, on a random sample of these worksites would provide valuable information on whether silicosis has, in fact, been controlled.

## Conclusions

Both the DEL's target of silicosis elimination by 2030 and the MHSC's milestone of no new cases in those first exposed after 2008 are laudable, but unlikely to be attained. The paucity of publically accessible information both with regard to levels of exposure and silicosis incidence means that solid evidence of progress to attainment is absent. However, the target and the milestone are both aspirational and we believe that they should be supported. Pragmatic methods need to be put in place to identify sources of new cases for targeted intervention, even if ascertainment is incomplete and it cannot be asserted that silicosis has been eliminated.

In the mining industry, concerted actions have been initiated to improve exposure assessment, e.g., to determine the silica percentage of the sampled dust of each filter and more importantly to control exposure, e.g., MOSH leading practices. Consequently, a decline in the silicosis incidence rates can be observed. However, it is uncertain whether this eventually will lead to elimination of silicosis, keeping in mind (i) the likely incomplete protection by the milestone RCS concentration of 0.05 mg/m^3^ TWA(8h), and (ii) issues related to under-diagnosis and under-reporting (illegal miners and “missing” workers either because they are employed by labor brokers or by return to labor-sending areas).

Accuracy and reliability of the RCS measurements, especially in the underground mines, should be improved. This can be achieved for example by supervision of gravimetric sampling processes during personal RCS measurement or the use of smart pumps that include an accelerometer and at-depth calibration of sampling pumps instead of on the surface. With the current knowledge on the health-relevance of small particles, an appropriate risk assessment should take these particles into account. We therefore recommend the inclusion of either size-selective sampling of the aerosols or additional size characterization of the particles collected on the cyclone's filter, by using laser light scattering for instance.

Identification of high-exposed occupations/job titles or tasks can be improved by (i) extension of the number of measurements for example by deploying (relatively) low-cost monitors and (ii) by introducing adopting more sophisticated data analysis methods, such as Bayesian statistics integrated in tools such as Expostats, and extension of the data set by pooling the data for a HEG over a year. Analysis of within- and between-worker variances can support this identification and might result in more adequate exposure grouping of workers.

In the non-mining sector, baseline data regarding both exposure levels and silicosis incidence rates are basically lacking. Therefore, a base-line study is recommended. However, keeping in mind limited resources, a prioritization of industries is key. In addition, the exposure assessments should provide sufficient information to identify high risk occupations and/or tasks, but even more importantly to target the development of interventions to control exposures. The development of an easy accessible exposure control repository comprising of activity-specific exposure control options could assist non-experts with selection of appropriate exposure controls. Such a repository for example as provided by OSHA (49, 64) should not only provide information on efficacy but also costs and feasibility issues regarding implementation.

For both mining and non-mining industry, identification of the major source of exposure and exposure pathway analysis are pivotal to the development and the sustainable implementation of dust prevention methods is key to eliminate silicosis.

Regard to the monitoring of cases of silicosis, both the DMRE and DEL have reporting systems that could give effect to a preventive strategy, since there is the potential to link sentinel cases to potentially problematic workplaces for targeted dust control interventions. A consideration in the link between disease occurrence and prevention is, however, the typically long latent period between first exposure and disease onset; high risk exposures may have occurred in the distant past and investigations of current workplace conditions may be unrewarding in preventing future cases. For this reason, silicosis cases with onset of disease after short durations of exposure (≤10–15 years) should be prioritized. To be successful, intensive sustained efforts to encourage the reporting of cases are needed, along with effective actions in response to sentinel cases. Data on disease incidence and preventive actions need to be communicated to tripartite occupational health and safety bodies and to the public.

## Data Availability Statement

The non-mining data analyzed in this study was obtained from the Department of Employment and Labor, with the restriction that no third party is permitted to read, copy, or use the data. Requests to access these datasets should be directed to Mrs. Bulelwa Huna (Bulelwa.Huna@labour.gov.za). Other data supporting the conclusions of this article are available on request to the corresponding author.

## Author Contributions

DB and DR participated in the work intellectually, took responsibility for the content of this article and read and approved the final manuscript. DB wrote the first draft and analyzed the exposure data. DR wrote the sections on silicosis.

### Conflict of Interest

The authors declare that the research was conducted in the absence of any commercial or financial relationships that could be construed as a potential conflict of interest.

## Footnotes

^1^https://www.mhsc.org.za/mining-legislation

^2^http://www.expostats.ca/site/en/index.html

^3^https://www.mosh.co.za/dust/leading-practices

^4^https://www.groundup.org.za/article/mines-report-progress-prevention-deadly-silicosis/

^5^https://www.fin24.com/Companies/everything-you-need-to-know-about-the-r5bn-silicosis-settlement-as-mining-companies-prepare-to-pay-out-20190803

^6^https://www.groundup.org.za/article/mines-report-progress-prevention-deadly-silicosis/
